# Use of photosensitising diuretics and risk of skin cancer: a population-based case–control study

**DOI:** 10.1038/sj.bjc.6604686

**Published:** 2008-09-23

**Authors:** A Ø Jensen, H F Thomsen, M C Engebjerg, A B Olesen, H T Sørensen, M R Karagas

**Affiliations:** 1 Department of Clinical Epidemiology, Aarhus University Hospital, Aarhus, Denmark; 2 Department of Dermatology, Aarhus University Hospital, Aarhus, Denmark; 3 Department of Community and Family Medicine, Section of Biostatistics and Epidemiology, Dartmouth Medical School, Lebanon, NH, USA

**Keywords:** diuretics, photosensitising reaction, skin cancer, epidemiology, risk

## Abstract

Diuretics have photosensitising properties. However, little is known about how these diuretics affect the risk of skin cancers. In North Jutland County, Denmark, we investigated whether the use of photosensitising diuretics was associated with an increased risk for developing basal cell carcinoma (BCC), squamous cell carcinoma (SCC) and malignant melanoma (MM). From the cancer registry, we identified primary cases of BCC, SCC and MM during the period of 1989–2003. We selected four population controls for each case from the Danish Civil Registration System, matched on age and gender. Prescriptions for photosensitising diuretics before cancer diagnosis were ascertained in the county's Prescription Database. We used conditional logistic regression to compute incidence rate ratio (IRR), controlling for the chronic medical conditions and for the previous use of oral glucocorticoids. We found an increased risk of SCC (IRR of 1.79 (95% confidence interval (CI): 1.45–2.21)) and MM (IRR of 1.43 (95% CI: 1.09–1.88)) among users of combined amiloride and hydrochlorothiazide therapy. An increased risk of MM (IRR of 3.30 (95% CI: 1.34–8.10)) was found among users of indapamide. We found little associations with risk of BCC. Our findings provide evidence that the use of some photosensitising diuretics is associated with an increased risk for SCC and MM.

Most diuretics are photosensitising, including loop diuretics (bumetanide (Frishman *et al*, 2002) and furosemide (Moore, 2002)), sodium-saving diuretics (spironolactone (Schwarze *et al*, 1998) and amiloride (Thestrup-Pedersen, 1987)) and thiazides (hydrochlorothiazide (Thestrup-Pedersen, 1987), bendroflumethiazide (Robinson *et al*, 1985; Diffey and Langtry, 1989; Johnston, 2002) and indapamide) with reports on adverse reactions of sun burns among users of these diuretics. In both Denmark (Thestrup-Pedersen, 1987) and England (Addo *et al*, 1987; Diffey and Langtry, 1989), similar reactions have been reported to occur specifically among those exposed to the combination of amiloride and hydrochlorothiazide treatment.

There is evidence that a photosensitising reaction followed by sun exposure may enhance the risk of sunburns and photo damage that increases risk of skin cancers (Stern, 1998). The mechanism of drug-induced photosensitivity causing cancer is well-known from psoralens combined with ultraviolet light-A (PUVA) therapy, which also increases the risk of squamous cell carcinoma (SCC) (Stern *et al*, 1984, 1998; Lindelof *et al*, 1991; Stern and Laird, 1994) and malignant melanoma (MM) (Stern, 2001). Although sun burns as a side effect to these diuretics may arise through the same mechanism as PUVA treatment, data on the photocarcinogenic potential of these drugs are limited. To date, only one recent formal case–control study from the United States investigated whether the use of photosensitising medications (which included diuretics) was associated with risk of non-melanoma skin cancers (NMSCs). On the basis of self-reported drug history, they found an increased relative risk of basal cell carcinoma (BCC) of 1.5 (95% confidence interval (CI): 1.0–2.4) and of SCC of 1.8 (95% CI: 1.1–3.2) if exposed to any of these drugs (Karagas *et al*, 2007). However, recall bias (Rothman, 2002) could not be excluded as an explanation for their findings.

In light of the common use of photosensitising diuretics, we conducted a large population-based case–control study in Denmark, examining whether the use of photosensitising diuretics increases the risk of BCC, SCC and MM.

## Materials and methods

We conducted this population-based case–control study in North Jutland County, with a population of about 500 000 inhabitants (approximately 9% of the Danish population). A unique 10-digit civil registry number assigned to all Danish residents enabled us to link national data on cancer incidence, prescription drugs and hospital discharge diagnoses.

From the Danish Cancer Registry (DCR), we identified all the patients registered with a first primary diagnosis of BCC, SCC or MM in North Jutland County from 1989 to 2003. The DCR, founded in 1943, records primary cases of cancer on a nationwide basis since 1943 with a high degree of accuracy (Storm *et al*, 1997). Data in the DCR include cancer type, site, morphology and date of diagnosis. Tumours in the DCR are coded according to a modified Danish version of the seventh revision of the International Classification of Diseases (ICD-7). Since 1978, tumours have been additionally classified according to the first version of the International Classification of Diseases for Oncology (ICD-O-1), which includes a four-digit code for tumour morphology.

We used ICD-7 codes 1910–1919 to identify all BCC and SCC cases registered in the County of North Jutland during the period 1989–2003. For MM, we used ICD-7 codes 1900–1909. For BCC, we included only patients with the following ICD-O-1 morphology codes: 80903, 80913, 80923, 80933 and 81233. For SCC, we included only patients with the ICD-O-1 codes 80513, 80703, 80713, 80743, 80763, 80943 and 80953. We identified a total of 5964 BCC cases, 1129 SCC cases and 1151 MM cases.

The Civil Registration System contains information about vital status, date of death and the area of residence of all Danish residents, and is updated daily (Frank, 2000). Using the Civil Registration System, we selected approximately four population controls for each case. Cases and controls were individually matched by exact age, gender and area of residence based on risk set sampling (Wacholder *et al*, 1992) (i.e., the controls had to be alive, and at risk for a first skin cancer at the time, the corresponding case was diagnosed (the index date assigned to controls)). A total of 32 412 population controls were selected for the 8244 BCC, SCC and MM cases.

Data on diuretic prescriptions were obtained from the Prescription Database in North Jutland County (Nielsen *et al*, 1997). The database collects data on all prescriptions filled by ambulatory patients and forwards data on reimbursable medicines to the local regional Health Service section on a monthly basis. This Health Service, in turn, refunds 50–75% of costs. The Prescription Database was established in 1989 (with complete coverage since 1991) and includes the specific drug prescribed according to the Anatomical Therapeutical Chemical (ATC) classification system, the date the prescription was filled, the packaging size, the number of pills in each package and the amount of drug in each pill. The ATC codes for the photosensitising diuretics used in this study are presented in Appendix 1.

In an earlier work, we found that chronic medical conditions that could be treated with diuretics increased the risk of skin cancers, that is, chronic pulmonary diseases, connective tissue diseases, renal diseases, organ transplants, and solid and haematological cancers (Jensen *et al*, 2008). To control for the potentially confounding effects of these chronic medical conditions, we retrieved all hospital diagnoses recorded in the Danish National Registry of Patients for our study population from 1 January 1977 (the date the Danish National Registry of Patients was established) to 31 December 2003. Diagnoses are coded according to the ICD-8 system through 1993 and according to the ICD-10 system thereafter. The Danish National Registry of Patients includes 99.4% of all discharge records from Danish hospitals (Andersen *et al*, 1999) and, since 1995, encompasses outpatient and emergency room visits also. For our analyses, we classified the diagnoses of chronic diseases into six general categories (described in Appendix 2).

Further, we retrieved prescriptions for oral glucocorticoids from the Prescription Database (ATC codes in Appendix 1), as the use of these drugs has been associated with an increased risk of skin cancers (Karagas *et al*, 2001; Sorensen *et al*, 2004) and could be a potential confounder in our analysis.

### Statistical analyses

For each subject, we identified all prescriptions for photosensitising diuretics before the date of primary skin cancer diagnosis or index date in the matched control.

We initially examined any prescriptions for diuretics in a dichotomous model (i.e., any diuretic prescription *vs* no prescriptions before the index date) for each case group (BCC, SCC and MM *vs* controls). We then examined the individual diuretic drugs by demographic characteristics (age and sex), anatomic site of the tumour (head and neck *vs* other sites), prior diagnoses and a previous prescription for oral glucocorticoids. For the individual diuretic drugs, we fitted two conditional logistic regression models for matched pair analysis for each case group (BCC, SCC and MM *vs* controls) and prescription of diuretics. The first logistic regression model treated diuretics as a dichotomous variable. Amiloride and hydrochlorothiazide was most frequently given as combination therapy; therefore, we did not control for use of the other. We attempted to separate the effects of amiloride and hydrochlorothiazide by classifying prescriptions for ‘amiloride only’, ‘hydrochlorothiazide only’, ‘both amiloride and hydrochlorothiazide’ and ‘none of the drugs’ as reference category. To examine dose–response relations, we computed the total amount of prescribed drug before the index date by multiplying the package size, the number of pills in each package and the amount of drug in each pill. The total amount of drug dispensed was included in the model as a continuous (linear) variable with ‘never prescribed the drug prior to the index date’ as reference category. When this amount of drug could not be computed (for instance, due to missing information in the prescription database), the average amount dispensed for that particular drug was estimated. Average amounts were used for 0.1% of the prescriptions of furosemide, 7% of the prescriptions of bumetanide, 24% of the prescriptions of amiloride, 0.1% of prescriptions of spironolactone, 23% of the prescriptions of hydrochlorothiazide, 10% of the prescriptions of bendroflumethiazide and 0.2% of the prescriptions of indapamide. In all the models, we included the six chronic disease categories (as shown in Appendix 2), prior prescription of a glucocorticoid (yes, no) and prescriptions for the other photosensitising diuretics (yes, no) as confounding factors.

We also conducted analyses excluding prescriptions issued within 1 year and 5 years of the skin cancer diagnosis or index date to evaluate the possibility of heightened detection of skin cancers due to medical surveillance among those prescribed diuretics (i.e., surveillance bias (Rothman, 2002)). These analyses also might point to possible latency effects (i.e., the period of time from diuretic exposure to the development of skin cancer). In addition, we conducted analyses stratified on the anatomic site of the cancers (i.e., head and neck and other sites) to evaluate the effects of photosensitising diuretics by the level of sun exposure.

The non-reporting of diagnosed NMSC to the DCR has been estimated to range from 12 to 40% (Frentz, 1996; Jensen *et al*, 2007). Therefore, we conducted a sensitivity analysis (Fox *et al*, 2005a; Greenland, 2005) to explore the magnitude of the effects of the non-reporting of NMSC cases on our results. We expected that the non-reporting of NMSC cases could be differential between users and non-users of photosensitising diuretics; as NMSC is rarely fatal, in severely diseased patients (such as users of diuretics), clinicians could potentially deem these cancers trivial and thereby omit registration. We used the SAS macro written by Fox *et al* (2005b), which was adapted to perform conditional logistic regression.

This study was approved by the Danish Protection Agency (record no. 2004-41-4693). The statistical software packages R, version 2.4.1., and SAS, version 9.1 (SAS Institute Inc., Cary, NC, USA) were used for all statistical analyses.

## Results

### Subject characteristics

The median age was 69 years among cases with BCC, 77 years among cases with SCC and 59 years among cases with MM. More SCC tumours developed on chronically sun-exposed sites (63% on the head and neck) than BCC or MM tumours (54 and 17% on the head and neck, respectively). In addition, more cases than controls had a history of chronic pulmonary diseases, connective tissue diseases, renal diseases, organ transplants, and solid and haematological cancers. Also, more cases had a prior prescription for oral glucocorticoids compared with the controls (data not shown).

### Prescriptions for photosensitising diuretics as risk factors for developing skin cancer

A total of 1026 (32%) BCC cases had used diuretics compared with 7717 (32%) controls, yielding an incidence rate ratio (IRR) for BCC of 0.96 (95% CI: 0.90–1.03). Further, there were no clear associations with any of the individually examined drugs (Table 1). The risk estimates for BCC of the head and neck were comparable with those of the other anatomic sites (data not shown).

A total of 493 (44%) SCC cases had used diuretics compared with 1743 (39%) controls, yielding an IRR for SCC of 1.21 (95% CI: 1.04–1.40). Individually, the use of amiloride, hydrochlorothiazide and the combination of the two was elevated for SCC cases compared with controls (Table 2). Among those with a previous prescription for amiloride, the IRR was 1.80 (95% CI: 1.46–2.20) and for hydrochlorothiazide, the IRR was 1.58 (95% CI: 1.29–1.93). Owing to the small number of subjects who were exclusively prescribed either amiloride or hydrochlorothiazide (0.7 and 0.4%, respectively), it was difficult to compute their separate effects. On the basis of small numbers, an elevated IRR was observed for those prescribed amiloride only (IRR=2.26; 95% CI: 0.94–5.43), but not for those prescribed hydrochlorothiazide only (IRR=0.38; 95% CI: 0.15–0.97). The joint IRR was 1.79 (95% CI: 1.45–2.21) (Table 2). Among users of all the individual diuretics (except bumetanide and indapamide), we observed that the estimated risk of SCC increased linearly with increasing amounts of prescribed drug (Table 2). Further, we observed an increased risk of SCC among users of amiloride, hydrochlorothiazide and the two drugs combined as the period of time from drug exposure to development of cancer (i.e., latency) increased. The joint IRR increased to 1.89 (95% CI: 1.52–2.35) with 1-year latency and 1.97 (95% CI: 1.49–2.62) with 5-year latency. This trend was particularly pronounced among those prescribed amiloride alone (Table 2). Risk estimates for cases with head and neck SCC were similar to those with SCC tumours at other anatomic sites (data not shown).

A total of 312 (27%) MM cases had used diuretics compared with 1124 (24%) controls, yielding an IRR for MM of 1.19 (95% CI: 1.01–1.41). For MM, we also observed an increased risk among those individually prescribed amiloride and hydrochlorothiazide (IRR=1.39; 95% CI: 1.06–1.81 and IRR=1.32; 95% CI: 1.03–1.70, respectively). Again, there were few cases who were prescribed exclusively one of these drugs; but as with SCC, the IRR was increased for prescription of amiloride only (IRR=1.21; 95% CI: 0.39–3.74), but not for hydrochlorothiazide only (IRR=0.87; 95% CI: 0.45–1.68). For prescription of combined therapy, the IRR was 1.43 (95% CI: 1.09–1.88) overall and somewhat higher for head and neck tumours (IRR of 2.15; 95% CI: 1.19–3.88 (data not shown)) compared with other anatomic sites. For MM, we also found an increased risk associated with use of indapamide (IRR=3.30; 95% CI: 1.34–8.10) (Table 3). Further, we observed a trend of an increasing risk of MM among users of indapamide, as the period of time from drug exposure to development of cancer (i.e., latency) increased. The IRR increased to 3.85 (95% CI: 1.47–10) with a 1 or more year latency and 6.06 (95% CI: 1.78–21) with a 5 or more year latency (Table 3).

### Sensitivity analyses simulating the non-reporting of NMSC cases

The sensitivity analyses showed that if we adjusted for a differential underascertainment of NMSC cases from 5 to 45% (trapezoidal distribution) among those prescribed diuretics, the resulting risk estimates for NMSC among those who were prescribed diuretics increased by a factor varying from 1.1 to 1.5, depending on the diuretic type (data not shown).

## Discussion

We found an increased risk of SCC and MM among the users of diuretics. We found an individual association between the use of combined amiloride–hydrochlorothiazide therapy and the risk of SCC, and an association, albeit weaker, between this therapy and MM. We also found evidence of a possible relation between indapamide use and risk of MM. We observed a trend of increasing risk with amount of drug and length of time between prescription and diagnosis (i.e., latency).

The mechanism of drug-induced photosensitivity causing cancer is well-known from observations of patients treated with PUVA therapy. These patients have increased risks of SCC and MM (Stern *et al*, 1984, 1998; Lindelof *et al*, 1991; Stern and Laird, 1994; Stern, 2001). It is of further interest to note that findings among those PUVA-treated patients show a clear dose–response relationship and latency effect on SCC risk (Stern *et al*, 1998). Similarly, we found an increased risk of SCC with increasing amount of prescribed amiloride and hydrochlorothiazide. This was particularly true for amiloride, which has a maximal absorbance in the UV-A spectrum (Moore, 2002). In contrast, we detected an effect of users of indapamide on MM risk, and this drug has a maximal absorbance in the UV-B spectrum (Davis *et al*, 1979). This may indicate a difference in wavelengths responsible for the distinct histologic types of skin cancer. Furthermore, only UV-B has been found to induce MM in transgenic mice (Larue *et al*, 1992).

In contrast, we did not find the same convincing association with use of photosensitising diuretics and risk of BCC. This is consistent with the studies relating sun exposure and BCC risk that indicate that the time between the carcinogenic UV exposure and the development of BCC is longer than that for SCC and that younger persons may be more susceptible to these effects than older persons (Gallagher *et al*, 1995).

The validity of our estimates depends on several factors. First, the uniformly organised Danish health-care system allows a true population-based design. Data on photosensitising diuretics were collected prospectively and independent of the cancer data, limiting the potential for recall bias found in interview-based studies on the same topic (Karagas *et al*, 2007). Moreover, our approach included assessment of the potential effects of a surveillance bias by excluding the use within 1 year of the cancer diagnoses; we found this bias to be slight. Finally, we considered the possibility that other factors may be confounding the association observed with photosensitising diuretics, and we were able to control prescriptions of glucocorticoids and a prior history for selected chronic diseases.

Among the limitations of our study was the reliance on prescription data and, specifically, the use of filling of prescriptions as a surrogate for actual using of the drugs (non-adherence). This could lead to misclassification of some non-users as users, potentially biasing our results towards unity. Still, the users had redeemed and paid for the drug; therefore, this bias is likely to be small. Another source of bias is that the records of NMSC may be incomplete in the DCR (Frentz, 1996; Frentz and Olsen, 1999). As NMSC is rarely fatal, in severely diseased patients, such as diuretic users, clinicians potentially could deem the NMSC trivial and therefore omit registration (differential registration). As shown by the sensitivity analyses, this potential underascertainment of NMSC cases using diuretics in our case–control study could have led to a substantial underestimation of the overall effect of diuretics on the risk of SCC and BCC.

As a register linkage study, we did not have information about skin phenotypes (i.e., skin sensitivity to sunlight and sun exposure). It is conceivable that patients with a sun-sensitive phenotype take more precaution when warned about the sun-sensitising effects of diuretics. If so, this could potentially underestimate the risk association, we observed. Another limitation is that some of the drug effects, that is ‘amiloride only’, ‘hydrochlorothiazide only’ and ‘indapamide’, as well as the drug-amount and latency trends were based on low proportions of cases. Therefore, these risk estimates had very limited statistical precision and should be interpreted with caution.

In conclusion, our large case–control study indicates a potential relation between commonly prescribed diuretics and risks of SCC and MM in the Danish population.

## Figures and Tables

**Table 1:** Use of photosensitising diuretics and risk of basal cell carcinoma

	**Basal cell carcinoma**				
	**Any prescription before diagnosis date**			**Prescriptions >1 year before diagnosis**	**Prescriptions >5 year before diagnosis**
	**Number of cases *N*=5964**	**Number of controls *N*=23856**	**IRR adjusted (95% CI)**	**IRR adjusted (95% CI)**	**IRR adjusted (95% CI)**
*Loop diuretics*					
Furosemide	830 (14%)	3422 (14%)	0.91 (0.83–0.99)	0.90 (0.82–0.99)	0.95 (0.83–1.09)
Linear increase per 10 000 mg			1.00 (0.99–1.01)		
Bumetanide	46 (0.8%)	211 (0.9%)	0.86 (0.62–1.19)	0.76 (0.53–1.09)	0.98 (0.59–1.63)
Linear increase per 10 000 mg			0.88 (0.16–4.66)		
					
*Potassium-saving diuretics*					
Amiloride	482 (8%)	1849 (7.8%)	1.04 (0.93–1.16)	1.03 (0.92–1.15)	1.11 (0.96–1.28)
Linear increase per 10 000 mg			1.13 (0.98–1.31)		
Spironolactone	145 (2.4%)	539 (2.3%)	1.06 (0.87–1.28)	1.10 (0.89–1.37)	1.20 (0.86–1.68)
Linear increase per 10 000 mg			1.04 (1.01–1.07)		
					
*Thiazide diuretics*					
Bendroflumethiazide	1069 (18%)	4291 (18%)	0.98 (0.90–1.06)	1.00 (0.91–1.08)	0.98 (0.87–1.10)
Linear increase per 10 000 mg			0.95 (0.80–1.13)		
Indapamide	24 (0.4%)	94 (0.4%)	0.99 (0.63–1.56)	0.94 (0.57–1.55)	0.90 (0.43–1.87)
Linear increase per 10 000 mg			0.88 (0.13–5.78)		
Hydrochlorothiazide	542 (9%)	2059 (8.6%)	1.05 (0.95–1.16)	1.05 (0.94–1.17)	1.10 (0.95–1.26)
Linear increase per 10 000 mg			1.02 (1.00–1.03)		
					
*Combined therapy*					
Amiloride only	24 (0.4%)	86 (0.4%)	1.18 (0.75–1.87)	0.92 (0.54–1.55)	0.93 (0.78–1.11)
Linear increase per 10 000 mg			a		
Hydrochlorothiazide only	84 (1.4%)	296 (1.2%)	1.14 (0.89–1.46)	1.17 (0.88–1.55)	0.97 (0.52–1.76)
Linear increase per 10 000 mg			a		
Amiloride and hydrochlorothiazide	458 (8%)	1763 (7%)	1.04 (0.93–1.16)	1.03 (0.92–1.16)	1.10 (0.95–1.27)
Linear increase per 10 000 mg			a		

CI=confidence interval; IRR=incidence rate ratio.

The reference group was different for each diuretic, as the reference group was ‘never users the particular diuretic under study’.

Conditional logistic regression was used to estimate IRRs and 95% CI, adjustments were made for a prior hospitalisation for selected chronic diseases and use of glucocorticoids.

a Not possible because of low proportions of cases or because of combined therapy.

**Table 2:** Use of photosensitising diuretics and risk of squamous cell carcinoma

	**Squamous cell carcinoma**				
	**Any prescription before diagnosis date**			**Prescriptions >1 year before diagnosis**	**Prescriptions >5 year before diagnosis**
	**Number of cases *N*=1129**	**Number of controls *N*=4516**	**IRR adjusted (95% CI)**	**IRR adjusted (95% CI)**	**IRR adjusted (95% CI)**
*Loop diuretics*					
Furosemide	235 (21%)	878 (19%)	0.94 (0.78–1.12)	0.91 (0.75–1.11)	0.92 (0.69–1.23)
Linear increase per 10 000 mg			1.01 (0.99–1.02)		
Bumetanide	9 (0.8%)	48 (1.1%)	0.71 (0.35–1.47)	0.63 (0.28–1.43)	0.89 (0.29–2.72)
Linear increase per 10 000 mg			4.78 (0.53–43)		
					
*Potassium-saving diuretics*					
Amiloride	162 (14%)	390 (9%)	1.80 (1.46–2.20)	1.90 (1.54–2.35)	1.98 (1.50–2.61)
Linear increase per 10 000 mg			1.31 (1.00–1.71)		
Spironolactone	38 (3.4%)	121 (2.7%)	1.11 (0.75–1.65)	1.00 (0.64–1.57)	0.90 (0.44–1.84)
Linear increase per 10 000 mg			1.00 (0.92–1.09)		
					
*Thiazide diuretics*					
Bendroflumethiazide	241 (21%)	913 (20%)	1.03 (0.86–1.22)	0.91 (0.76–1.10)	1.03 (0.79–1.34)
Linear increase per 10 000 mg			0.98 (0.71–1.34)		
Indapamide	10 (0.9%)	29 (0.6%)	1.20 (0.57–2.54)	1.10 (0.49–2.46)	1.02 (0.32–3.23)
			a		
Hydrochlorothiazide	159 (14%)	427 (10%)	1.58 (1.29–1.93)	1.67 (1.36–2.07)	1.92 (1.46–2.54)
Linear increase per 10 000 mg			1.03 (1.01–1.06)		
					
*Combined therapy*					
Amiloride only	8 (0.7%)	18 (0.4%)	2.26 (0.94–5.43)	2.51 (1.03–6.13)	3.42 (0.75–15.6)
Linear increase per 10 000 mg			a		
Hydrochlorothiazide only	5 (0.4%)	55 (1.2%)	0.38 (0.15–0.97)	0.29 (0.09–0.94)	1.33 (0.25–6.93)
Linear increase per 10 000 mg			a		
Amiloride and hydrochlorothiazide	154 (14%)	372 (8%)	1.79 (1.45–2.21)	1.89 (1.52–2.35)	1.97 (1.49–2.62)
Linear increase per 10 000 mg			a		

CI=confidence interval; IRR=incidence rate ratio.

The reference group was different for each diuretic, as the reference group was ‘the never users the particular diuretic under study’.

Conditional logistic regression was used to IRRs and 95% CI, adjustments were made for a prior hospitalisation for selected chronic diseases and use of glucocorticoids.

The linear increase per 10 000 mg prescribed drug translates to the average percentage increase per 10 000 mg of prescribed drug.

a Not possible because of low proportions of cases or because of combined therapy.

**Table 3:** Use of photosensitising diuretics and risk of malignant melanoma

	**Malignant melanoma**				
	**Any prescription before diagnosis date**			**Prescriptions >1 year before diagnosis**	**Prescriptions >5 year before diagnosis**
	**Number of cases *N*=1010**	**Number of controls *N*=4040**	**IRR adjusted (95% CI)**	**IRR adjusted (95% CI)**	**IRR adjusted (95% CI)**
*Loop diuretics*					
Furosemide	116 (10%)	487 (11%)	0.91 (0.72–1.16)	0.90 (0.69–1.16)	0.90 (0.63–1.29)
Linear increase per 10 000 mg			1.01 (0.99–1.03)		
Bumetanide	6 (0.5%)	29 (0.6%)	0.75 (0.29–1.90)	0.79 (0.28–2.23)	0.69 (0.18–2.61)
Linear increase per 10 000 mg			0.20 (0.00–202)		
					
*Potassium-saving diuretics*					
Amiloride	90 (8%)	272 (6%)	1.39 (1.06–1.81)	1.33 (1.00–1.77)	1.29 (0.89–1.86)
Linear increase per 10 000 mg			0.76 (0.50–1.17)		
Spironolactone	14 (1.2%)	67 (1.5%)	0.84 (0.46–1.54)	0.91 (0.46–1.79)	0.71 (0.26–1.93)
Linear increase per 10 000 mg			1.01 (0.94–1.09)		
					
*Thiazide diuretics*					
Bendroflumethiazide	168 (15%)	616 (13%)	1.08 (0.88–1.32)	1.06 (0.86–1.32)	1.01 (0.74–1.37)
Linear increase per 10 000 mg			1.11 (0.70–1.75)		
Indapamide	10 (0.9%)	12 (0.3%)	3.30 (1.34–8.10)	3.85 (1.47–10.1)	6.06 (1.78–20.7)
Linear increase per 10 000 mg			5.81 (0.07–464)		
Hydrochlorothiazide	98 (9%)	303 (7%)	1.32 (1.03–1.70)	1.30 (0.99–1.71)	1.24 (0.86–1.78)
Linear increase per 10 000 mg			0.99 (0.95–1.03)		
					
*Combined therapy*					
Amiloride only	5 (0.4%)	15 (0.3%)	1.21 (0.39–3.74)	1.10 (0.31–3.95)	2.56 (0.44- 14.9)
Linear increase per 10 000 mg			a		
Hydrochlorothiazide only	13 (1.1%)	46 (1.0%)	0.87 (0.45–1.68)	0.91 (0.43–1.92)	0.89 (0.26–3.03)
Linear increase per 10 000 mg			a		
Amiloride and hydrochlorothiazide	85 (7%)	257 (6%)	1.43 (1.09–1.88)	1.37 (1.02–1.83)	1.28 (0.87–1.86)
Linear increase per 10 000 mg			a		

CI=confidence interval; IRR=incidence rate ratio.

The reference group was different for each diuretic, as the reference group was ‘the never users the particular diuretic under study’.

Conditional logistic regression was used to estimate IRRs and 95% CI, adjustments were made for a prior hospitalisation for selected chronic diseases and use of glucocorticoids.

a Not possible because of low proportions of cases or because of combined therapy.

**Table A1 tbla1:** 

**Photosensitising diuretics/glucocorticoids**	**ATC codes**
Furosemide	C03CA01, C03EB01
Bumetanide	C03CA02, C03EB02
Amiloride	C03EA01
Spironolactone	C03DA01
Hydrochlorothiazide	C03EA01, C07BB02, C09BA01, C09BA02, C09BA03, C09BA05, C09DA06, C09DA04, C09DA01, C09DA07, C09DA02 and C09DA03
Bendroflumethiazide	C03AA01
Indapamide	C03BA11
Budenoside	A07EA06
Hydrocortisone	A07EA02, H02AB09
Prednisolone	A07EA01, H02AB06
Prednisone	H02AB07
Betamethasone	H02AB01
Methylprednisolone	H02AB04
Triamcinolone	H02AB08

**Table A2 tbla2:** 

**Disease category**	**Diseases**	**ICD-8**	**ICD-10**
Chronic pulmonary disease	Emphysema and chronic obstructive lung disease	490–493; 515–518	J40–J47; J60–J67; J68.4; J70.1; J70.3; J84.1; J92.0; J96.1; J98.2; J98.3
Connective tissue disease	Diffuse connective tissue disease, rheumatoid arthritis and other inflammatory polyarthropathies and polymyalgia rheumatica	712; 716; 734; 446; 135.99	M05; M06; M08; M09; M30–M36; D86
Moderate-to-severe renal disease	Glomerulonephritis, nephropathies and end-stage renal disease	403; 404; 580–583; 584; 590.09; 593.19; 753.10–753.19; 792	I12; I13; N00–N05; N07; N11; N14; N17–N19; Q61
Organ transplants	Heart, kidney, liver and bone marrow transplantation	Y9509	Z94
Any solid cancer except skin cancer and metastasis	Any solid cancer except skin cancer and metastasis	140–199, except 172 and 173	C00–C80, except C43–C44
Leukaemia and Lymphoma	Non-Hodgkin and Hodgkin lymphoma and multiple myeloma	200–207; 275.59	C81–C85; C88; C90–C96

## Appendix 1

The anatomical therapeutical chemical (ATC) codes for the diuretics and the glucocorticoids

[Table tbla1]

## Appendix 2

Translation of disease categories of chronic diseases into discharge diagnoses in ICD-8 and ICD-10

[Table tbla2]
